# Dataset of speech produced with delayed auditory feedback

**DOI:** 10.1016/j.dib.2025.111300

**Published:** 2025-01-14

**Authors:** Matthias Heyne, Monique C. Tardif, Alexander Ocampo, Ashley P. Petitjean, Emily J. Hacker, Caroline N. Fox, Megan A. Liu, Madeline Fontana, Vincent Pennetti, Jason W. Bohland

**Affiliations:** a Department of Communication Disorders, State University of New York at New Paltz, New Paltz, NY 12561, United States; b Department of Communication Science and Disorders, University of Pittsburgh, Pittsburgh, PA 15260, United States; c Institute of Plant Breeding, Genetics, and Genomics, University of Georgia, Athens, GA, United States; d Department of Bioengineering, University of Pittsburgh, Pittsburgh, PA, United States; e Center for the Neural Basis of Cognition, Pittsburgh, PA, United States

**Keywords:** Speech production, Speech motor control, Acoustic, Phonetics, Speech errors

## Abstract

Speakers use *auditory feedback* to monitor their speech output and detect any deviations from their expectations. It has long been known that when auditory feedback is artificially delayed by a fraction of a second, speech may be severely disrupted [[1], [2], [3]]. Despite the long history of using delayed auditory feedback (DAF) in experimental research on speech motor control, its effects remain relatively poorly understood. To our knowledge, there are currently no publicly available research datasets containing recordings of speech produced with DAF. Here we describe a large dataset of speech produced with DAF using modern experimental methods with systematic controls and varied speaking materials, including phonotactically legal, nonword syllable sequences and American English sentences. Auditory feedback latencies were tightly controlled and included a zero / minimal delay (∼12 ms), 150 ms, 200 ms, and 250 ms. The dataset includes simultaneous audio recordings from the microphone (production) and headphone (feedback) channels. It also includes recordings and annotations of reading passages and multiple other demographic and acoustic measures that serve as covariates of interest from each participant. The complete dataset, which is made available in two segments (one fully open access and one password restricted) includes speech audio recordings from 55 participants, 42 of whom completed a second session with similar testing materials. This dataset is valuable for researchers interested in theoretical aspects of speech sensory-motor control and for researchers interested in developing speech analysis tools.

Specifications Table

**Table:** 

Subject	Linguistics
Specific subject area	Speech motor control. The dataset addresses the effects of delayed auditory feedback on the control of speech output in neurotypical adults*.*
Type of data	Audio recordings (.wav): mono, 16-bit precision, 48 kHz sampling rate. TextGrids (.TextGrid): files formatted for Praat software [4] containing annotations of reading passage audio recordings. Tables (.xlsx): spreadsheets containing full specification of the stimulus conditions for each participant. Tables (.xlsx): spreadsheets containing participant-level demographic variables and extracted covariates of interest.
Data collection	Audio recordings were obtained in a sound-treated room using a head-worn omni-directional condenser microphone (Shure MX153) and/or a stand-mounted condenser microphone (Shure SM81 or Audio-Technica AT875R) attached to a USB audio interface (RME FireFace UFX+). Audio signals were digitized and saved using Audacity software and further processed offline. Demographic data were obtained through an in-person computer survey, and covariates of interest were extracted from experimental data (audio and other behavioral response data)*.*
Data source location	Data were collected in-person in the Speech Neural Systems Laboratory at the University of Pittsburgh (Pittsburgh, PA, USA)*.*
Data accessibility	Repository name: Open Science Framework (osf.io) Data identification number: DOI 10.17605/OSF.IO/VZ6UH Direct URL to data: https://osf.io/vz6uh/
Related research article	None*.*

## 1 Value of the Data

• The dataset represents a large (*N*=55 participants and over 33,000 experimental trials), modern sample of speech produced under delayed auditory feedback (DAF) conditions in neurotypical adults (ages 18-60). Though DAF is an experimental manipulation that has been used in speech research for more than 70 years [1], its effects remain relatively poorly understood. DAF leads to reduced speaking rates, changes in pitch and intensity, and distorted and disfluent speech. These effects highlight the importance of self-generated auditory feedback for fluent speech production. While speech under DAF has been extensively studied, the previous literature has typically used relatively small speech samples, heterogeneous speaking conditions and stimuli, and has lacked important controls. This dataset is, to our knowledge, the largest available database of speech produced with DAF and thus has value to researchers interested in sensorimotor control of speech.
• The data were obtained under experimental conditions involving the production of typical English sentences as well as non-word, phonotactically legal syllable sequences. These two broad stimulus categories provide for different perspectives on the sensorimotor processes underlying speech. Non-word syllable sequences (including 3- and 4-syllable sequences) highlight aspects of speech motor control that are largely isolated from many cognitive and linguistic factors (e.g., long-term memory of words, lexical access and syntactical processes), but which place high demands on phonological working memory. English sentences offer more naturalistic, continuous speech conditions that vary in timing and prosody. Together, these conditions enable examination of how DAF gives rise to differential profiles of speech changes under variable speaking demands.
• The dataset includes speech produced with different, tightly controlled feedback latencies (“zero” or minimal delay, 150 ms, 200 ms, 250 ms) in both broad speaking tasks (non-word syllable sequences and sentences). The availability of data across delays allows for parametric examinations of the effects of DAF (e.g., as a function of latency alone, or with respect to participant speaking rate).
• The dataset includes many productions from each speaker (typically 128 trials of 3-syllable sequences, 128 trials of 4-syllable sequences, and 100 trials of sentences per speaker per session). Each speaker was tested across all conditions, allowing for within-subject comparisons of the effects of DAF as well as the ability to assess individual variability. The dataset includes a second session for 42 of 55 speakers, thus allowing for test-retest assessments. It also includes typical speech samples (reading passages) that have been annotated and analyzed, and other measures (e.g., perceptual acuity, spontaneous speech synchronization index; [5]) from each speaker. These additional measures may be of interest in isolation, and/or researchers may have interest in testing associations between these measures and the effects of DAF.
• The data provide a rich source of high-quality speech recordings that can be used as a basis for developing algorithms for speech error detection, recognition of atypical, distorted speech (e.g., as might be encountered in clinical populations), and/or developing explanatory computational models related to speech motor control.

## 2 Background

Our overall objective in acquiring this dataset was to understand how DAF disrupts speech motor sequencing, leading to errors in speech output. During speech, an auditory signal is transmitted not only to the intended listener, but also back to the speaker as *auditory feedback*. This signal is critical for learning and maintaining sensorimotor mappings needed for effective speaking, and for online monitoring of speech for errors. Real-time alterations of auditory feedback drive changes to the speech motor controller (e.g., eliciting compensatory responses during pitch [6] or formant-shifted feedback [7]). Such effects can be partly accounted for by feedback-based models of speech production. However, DAF causes changes to speech at different levels (e.g., rate reductions, pitch and intensity changes, serial order errors) that are less well understood and less well accounted for by existing models [8]. To help address this gap in understanding, we sought to systematically determine the effects of DAF on speech sequencing using modern experimental methods and a range of different stimuli. We also collected additional speech-related measures from each participant as covariates of interest. We hope that public release of this dataset will promote further investigation and help advance theoretical understanding of this long-studied experimental manipulation.

## 3 Data Description

The dataset [9] is made available in two segments, based on permissions obtained from research participants: (1) a fully open, unrestricted dataset (*Open Access Dataset*), and (2) a restricted, password-protected dataset made available only to registered researchers (*Restricted Dataset*). Each segment contains data with a common overall structure and format as described below.

The top-level folder structure (repeated for both dataset segments) describes individual participants and includes a Microsoft Excel spreadsheet (.xlsx file) containing participant-level variables. The structure of this participant-level table is described in Table 1. The top-level folder also contains .xlsx files that describe (i) the number of participants, sessions, and files available, and (ii) the combined set of researcher notes related to individual trials or data files (e.g., files to omit from analyses or technical concerns with one or more experimental trials). A set of .csv files also provide data dictionaries with further explanation of individual variables (column headings) found in the provided data files.

**Table 1:** Participant-level variables found in the files participants_openaccess.xlsx and participants_restricted.xlsx. Several variables (columns) are repeated across sessions (indexed by variable *i* in the table below, *i*=1 or 2) and across reading passages (indexed by variable *j*=1 or 2). For participants who did not complete a second session, entries related to that session are left blank (missing values) in the spreadsheet. The methods used to estimate these variables are described in the *Experimental Design, Materials and Methods* section.

Column Name (Variable)	Description
SubjectID	Unique alphanumeric subject identifier (e.g., NSFDAF001)
Age	Age at time of testing in years
Sex	Self-reported biological sex at birth (male, female, intersex, other)
Gender	Self-reported gender (optional free text description)
Languages	Languages the participant has used, in order of proficiency
Lang_Prof	Self-reported proficiency with reported languages used
Lang_Age	Approximate age (in years) at which participant began learning reported languages used
Sess<i>_HSR<j>_Passage	“CATERPILLAR” or “GRANDFATHER” to indicate which passage was used to probe habitual speaking rate (HSR) for the *jth* reading passage in Session *i*
Sess<i>_HSR<j>_SpeechRate	Speech rate (syllables per second) estimated from *jth* first reading passage recording in Session *i*
Sess<i>_HSR<j>_ArticRate	Articulation rate (syllables per second excluding pauses) estimated from the *jth* reading passage recording in Session *i*
Sess<i>_HSR<j>_MedianF0	Median voice fundamental frequency (in Hertz) across all voiced segments estimated from the *jth* reading passage recording in Session *i*
Sess<i>_HSR<j>_IQRF0	Interquartile range of voice fundamental frequency (in Hertz) across all voiced segments estimated from the *jth* reading passage recording in Session *i*.
Sess<i>_HSR<j>_IQRIntensity	Interquartile range of speech intensity (in dB SPL) estimated from the *jth* reading passage recording in Session *i*.
Sess<i>_HSR<j>_VSA	Working vowel space area estimated from the *jth* reading passage recording in Session *i* in units of squared kilohertz.
Sess<i>_F1_JND	Just noticeable difference (percent change in first formant frequency value in Hertz) obtained in a perceptual acuity experiment in Session *i*.
SSS_Index1	Spontaneous Speech Synchronization (SSS) Index obtained from the first block of the SSS Task. Score ranges from 0 to 1.
SSS_Index2	Spontaneous Speech Synchronization (SSS) Index obtained from the second block of the SSS Task. Score ranges from 0 to 1.

**Fig. 1:**
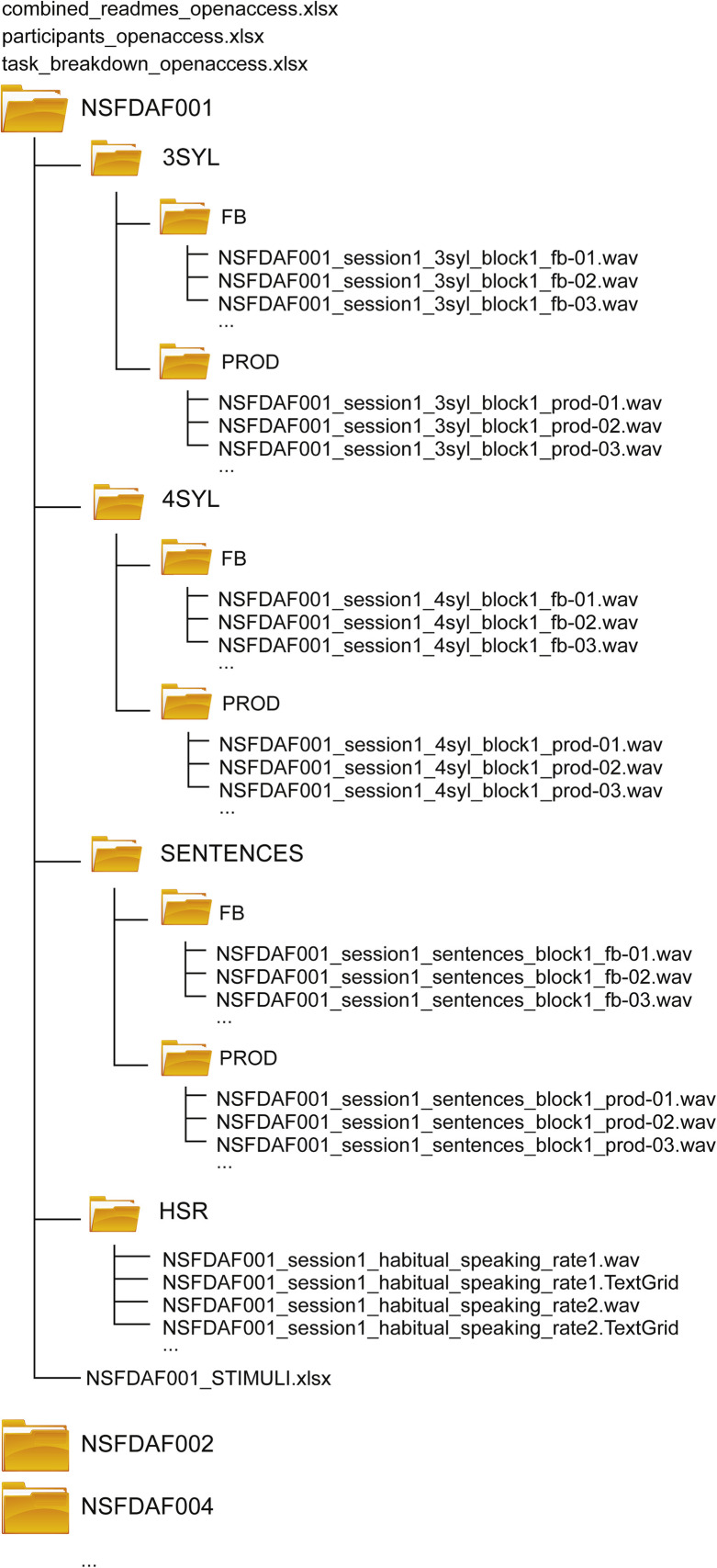
Folder and file structure found within each segment (*Open Access* and *Restricted*) of the dataset. Participant-level folders contain folders for each task, with subfolders for feedback (FB) and production (PROD) recordings (except in the case of the habitual speaking rate / reading passage task). A .xlsx spreadsheet is found in each participant's root folder and describes detailed experimental conditions for each trial. Spreadsheets at the overall root level for each dataset segment provide participant level variables, combined “readmes” (notes on any technical issues), and a breakdown of the contents of the provided data by task.

All audio data from an individual participant are found within a folder named using a unique, non-identifiable alphanumeric code (e.g., *NSFDAF001*). These participant-level folders each contain a .xlsx spreadsheet describing stimuli encountered by that participant (see Table 2). Within each folder, the following subfolders are found, containing data related to different experimental tasks (see Fig. 1):

• **3SYL:** audio recordings of microphone (PROD) and headphone (FB) signals for 3-syllable sequence stimuli.
• **4SYL:** audio recordings of microphone and headphone signals for 4-syllable sequence stimuli.
• **SENTENCES:** audio recordings of microphone and headphone signals for sentence stimuli.
• **HSR:** audio recordings and annotations (TextGrids) of reading passage recordings aimed at measuring habitual speech rate (HSR) and other speech metrics.

**Table 2:** Example excerpt from a participant-level stimulus conditions worksheet showing the first 10 trials of one block of the *SENTENCES* task from Session 1. The *TIMIT* column encodes a path to the specific audio file used as an audio exemplar in the TIMIT speech corpus [10]^1^. Similar stimulus worksheets are provided for the *3SYL* and *4SYL* tasks.

session	block	trial	stimulus	Delay	TIMIT
1	1	1	Fill that canteen with fresh spring water.	0	TIMIT/TRAIN/DR4/FJWB1/SX75.wav
1	1	2	Would a tomboy often play outdoors?	0	TIMIT/TRAIN/DR2/MWSB0/SX186.wav
1	1	3	Steve wore a bright red cashmere sweater.	200	TIMIT/TEST/DR4/MJDM1/SX275.wav
1	1	4	Continental drift is a geological theory.	150	TIMIT/TRAIN/DR7/MDLR0/SX63.wav
1	1	5	Those who teach values first abolish cheating.	250	TIMIT/TRAIN/DR6/MABC0/SX421.wav
1	1	6	Young people participate in athletic activities.	250	TIMIT/TRAIN/DR4/MRAB1/SX38.wav
1	1	7	Youngsters love common candy as treats.	150	TIMIT/TEST/DR4/FEDW0/SX94.wav
1	1	8	The drunkard is a social outcast.	200	TIMIT/TRAIN/DR2/MDEM0/SX158.wav
1	1	9	Did you eat yet?	250	TIMIT/TRAIN/DR2/MRLJ0/SX70.wav
1	1	10	Porcupines resemble sea urchins.	200	TIMIT/TRAIN/DR4/MMBS0/SX71.wav

Within the *3SYL, 4SYL*, and *SENTENCES* folders are subfolders:

• **FB:** contains recordings from the “feedback” channel (e.g., signals that were presented over headphones).
• **PROD:** contains recordings (synchronized with correspondingly named files in the FB subfolder) from the “production” channel (e.g., signals that were recorded from the participant microphone).

Across all three tasks, paired audio files (across FB and PROD folders) are provided for each individual trial. All audio files are mono, 16-bit precision WAVE audio files (.wav), sampled at 48 kHz. The file naming convention is as follows:

**<*subject_id*>_session<*session_num*>_<*task*>_block<*block_num*>_<*channel*>-<*trial_num*>.wav,** where bracketed variables are replaced with values as indicated below:

• ***subject_id:*** unique alphanumeric code for each participant (same as folder name).
• ***session_num:*** either 1 or 2, indicating if the data were recorded in the first or second session that participant attended.
• ***task:*** “3syl,” “4syl,” or “sentences.”
• ***block_num:*** integer indicating which block of the task (1 or 2) the recording was from within the session.
• ***channel:*** “fb” or “prod” indicating whether the channel was the feedback (headphone) signal or the production (microphone signal) on that trial.
• ***trial_num:*** running integer index with leading zeros (01, 02, …) of the sequential trial number within a given block of a task.

Occasionally, one or more audio files are missing due to technical problems. Such files are replaced with a .txt file with the same file prefix, which includes a brief note describing any relevant issues.

The *HSR* folder (within each participant's root folder) contains recorded audio from ∼1-2 minute duration, standardized reading passages, and aligned Praat-compatible *TextGrid* files with the same file prefix. TextGrid files contain interval-based annotations of the audio at the word and phone levels in two tiers. In the phone tier, the code “sil” is used to indicate silence, and “sp” is used to indicate a short pause. These files are named as:

**<*subject_id*>_session<*session_num*>_habitual_speaking_rate<*trial_num*>.<*extension*>,** with the italicized variables replaced as noted above for other tasks, and with the addition of <*extension*> taking the value of *.wav* or *.TextGrid*.

Table 3 describes the scale of the dataset and the distribution of data across different speaking tasks, for both the Open Access and Restricted segments. Across both segments, the dataset includes more than 66,000 audio files.

**Table 3:** Overall extent of the dataset, broken down by task and segment (Open Access vs. Restricted). These numbers represent all valid trials (i.e., excluding trials with known technical issues). The total number of audio files includes both feedback and production audio files for each valid trial.

	# speakers	# sessions	# 3SYL trials	# 4SYL trials	# SENTENCES trials	# HSR recordings	Total number of audio files
Open Access Dataset	23	40	4952	4898	4000	80	27780
Restricted Dataset	32	57	7004	6820	5650	114	39062
Total	55	97	11956	11718	9650	194	66842

The *Open Access Dataset* (DOI 10.17605/OSF.IO/VZ6UH) is licensed under Creative Commons Attribution 4.0 International Public License (CC-BY), allowing users to distribute, remix, or adapt materials if attribution is given to the creators. Access to the additional *Restricted Access Dataset* can be requested directly through the OSF web site, which will then be reviewed and, if appropriate, approved by the corresponding author. *Restricted Access Data* must be protected and may not be further redistributed.

## 4 Experimental Design, Materials and Methods

**Design.** The overall motivation for the study leading to this dataset was to systematically determine how manipulations to auditory feedback drive changes in serial speech output. This was an experimental research study using quantitative methods. It used a within-subjects design, where each participant was exposed to all experimental conditions. This design, whereby each subject serves as their own control, was chosen to maximize sensitivity to the effects of the primary independent variable (i.e., the feedback alteration). By obtaining a large set of speech samples from each participant, we aimed to enable meaningful inferences about individual-specific effects and intersubject variability. We also made several additional measurements and collected demographic information from participants to be used primarily as covariates of interest at the participant level.

**Participants.** All participants self-reported as native American English speakers, ages 18-60, with healthy, normal voice, speech, and hearing. Participants were excluded if they reported a previous history of a diagnosis of a speech, language, or hearing disorder; previous treatment for a speech, language, or hearing disorder; or a history of neurological disease or stroke. Participants were also excluded if they failed a hearing screening administered at the onset of study enrollment (see below).

56 participants passed the hearing screening and completed Session 1. Data from one of these participants were excluded from the dataset due to difficulties completing the tasks as instructed, leaving a sample size of *N=*55 (44 women), 42 of whom completed Session 2. 23 of these participants gave permission for their data to be shared in an open-access database, and 32 participants gave their permission for their data to be shared in a restricted (password-protected) database. The study was approved by the University of Pittsburgh Institutional Review Board. Participants were financially compensated for their time.

**Audiometric screening.** Prior to Session 1, a brief ∼10-minute pure tone hearing screening was administered in a double-wall sound-treated booth using an Otometrics Madsen Astera audiometer and foam insert earphones. Screening was performed at 25 dB HL for octave frequencies between 250 Hz and 8 kHz. 8 participants gave informed consent but failed this hearing screening and were excluded from the study (these participants are not represented in this dataset and are not included in the *N*=55 sample size).

**Demographic survey.** Upon passing audiometric screening in Session 1, participants were asked to complete a brief survey to provide demographic information. In this dataset, we provide participants’ self-reported age at testing and assigned sex at birth (choices were female, male, intersex, or other; an option to opt-out of answering was also provided but not used by any participants). We also asked participants the free response question, “How do you currently describe your gender identity (leave blank if you prefer not to answer)?” These responses are also included in the dataset. We also asked participants about their language experience. Participants were asked to list, in order of proficiency, any languages they have used. For each self-reported language used, they were asked to rate (i) their proficiency with that language using a 5-point Likert scale (1 = not at all proficient; 2 = slightly proficient; 3 = somewhat proficient; 4 = moderately proficient; 5 = extremely proficient), and (ii) the approximate age (in years) at which they began learning that language (with zero indicating “from birth”). These data are also included in the participant-level variables in the dataset.

**Fig. 2:**
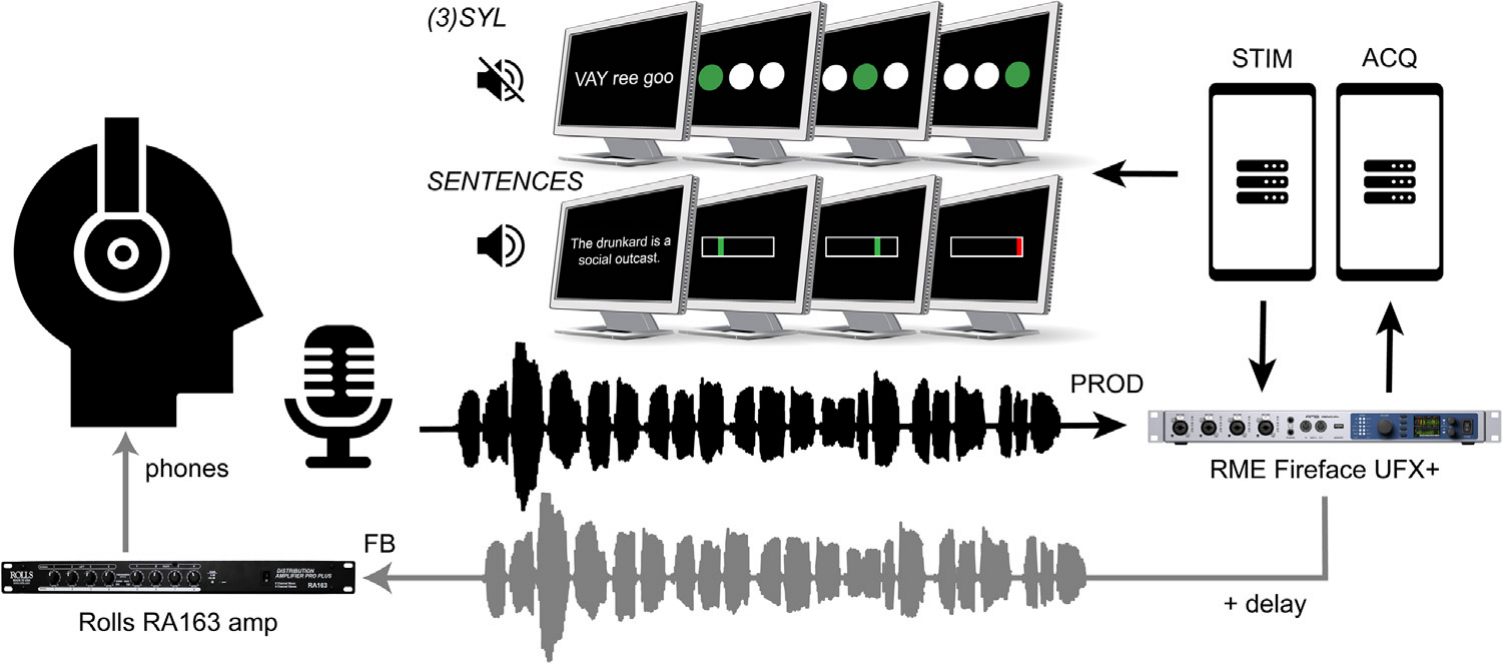
Experimental setup for recording speech under delayed auditory feedback. Top center depicts visual stimuli provided to participants during performance of 3SYL and SENTENCES tasks (4SYL mirrored 3SYL but with four circles providing a visual metronome signal). Syllable sequences were provided without auditory exemplars, while sentences were both heard and read by participants.

**Experimental procedures.** Primary experimental testing was performed inside a sound-treated room. Participants sat in a comfortable chair and viewed stimuli on a 24-inch monitor (VIEWPixx/EEG, VPixx Technologies) placed ∼1.5 m in front of them. For most tasks, participants were fitted with a flexible, over-the-ear omnidirectional condenser microphone (Shure MX153) positioned at ∼7 cm distance and 45° from the corner of the mouth. Auditory feedback was delivered via closed-back headphones (Sennheiser HD 280 Pro, Sennheiser Group). Fig. 2 illustrates the basic experimental setup.

Stimulus presentation was controlled using MATLAB and the Psychophysics Toolbox [11], running on a Windows-based workstation. This workstation was connected to an external audio device (RME Fireface UFX+; RME Audio) via USB 3.0, with hardware sampling rate set to 48 kHz and buffer size set to 256 samples. Signals were relayed from the audio device to the participants’ headphones via an amplifier (Rolls RA163; Rolls Corporation). The audio system was calibrated to achieve a net +5 dB gain between the microphone signal and headphone signal to partially mask bone-conducted feedback. The microphone and concurrent headphone signals were transmitted via ADAT digital optical cable to a separate Windows-based workstation equipped with a high-fidelity, multi-channel PCI audio card (RME HDSP 9632) and digitized at 48 kHz using Audacity software (http://audacityteam.org). The timing of auditory feedback (see Speech DAF Tasks below) was controlled using PsychPortAudio, a software sound interface that uses Audio Stream Input / Output (ASIO) to obtain high temporal precision and low latency sound playback. For the zero (minimal) delay case, a special *Direct Input Monitoring* mode was used, resulting in a ∼12 ms delay between the microphone and headphone signals, measured using the method described by Kim et al. [12].

*Reading passages (HSR)*. In each session, participants were first asked to read aloud one of two phonetically balanced reading passages. The two reading passages used were: (1) The Caterpillar Passage [13] during Session 1, and (2) The Grandfather Passage [14] during Session 2. The purpose of these tasks was to obtain measures of habitual speaking rate (HSR) and other acoustic phonetic features. Participants were instructed to familiarize themselves with the passage (presented on screen) by silently reading it through twice. Then, they were prompted to read the passage aloud “as if you were telling the story to me, at your comfortable speaking rate and usual conversational volume.” Each passage was repeated and recorded twice. This task was performed without headphones and was recorded using a condenser microphone (Shure SM81 or Audio-Technica AT875R) mounted on a microphone stand placed next to the seated participant.

*Speech DAF tasks.* Participants produced nonword syllable sequences and sentences under different auditory feedback delays. Speech was produced from memory following the presentation (and subsequent removal) of a stimulus. In all experimental conditions, the microphone signal (speech) and headphone signal (feedback) were concurrently recorded as described above.

*Nonword syllable sequences (3SYL and 4SYL).* Three-syllable (*3SYL*) and four-syllable (*4SYL*) CV, CVC, and VC (C=consonant, V=vowel) sequences were assembled from a closed set of vowels (/a/, /i/, /u/, /eɪ/) and consonants (/b/, /d/, /g/, /m/, /v/; /r/ in onset position only). Stimuli for each trial were selected pseudo-randomly from the full set of combinations, sampling consonants and vowels in equal numbers without repetition of sounds in adjacent syllables. On each trial, one sequence was presented orthographically (e.g., “VAY ree goo”; “BEE vay ROO gahm”) on the monitor for 2 s (*3SYL* case) or 3.5 s (*4SYL* case), with stress assigned to one syllable (*3SYL* case) or two syllables (*4SYL* case; stress always on 1^st^/3^rd^ or 2^nd^/4^th^ syllables) as indicated by upper case letters.

For the *3SYL* and *4SYL* tasks, on each trial, the participant was asked to memorize and prepare to repeatedly produce the memorized sequence when cued. The cue to speak was a visual change, in which the stimulus was removed and replaced by a “visual metronome” (a series of circles that sequentially changed colors; see [8]) that encouraged subjects to produce steady, consistent, rhythmic speech, with a rapid target rate of 5 Hz (200 ms between syllable onsets). The production / recording period was 3200 ms for each trial. Subjects were instructed to overtly produce the most recently presented sequence repeatedly throughout the production period while receiving auditory feedback through headphones and to pace their utterances approximately to the metronome. Subjects were directed to attempt to maintain the metronome rate as closely as possible, even to the detriment of “correct” speech output if necessary. These instructions were intended to combat the natural tendency for speakers to reduce their speech rate under DAF. Each experimental block consisted of 62 trials, each involving the presentation and production of one sequence with auditory feedback delays chosen pseudorandomly in equal proportions from (0, 150, 200, 250 ms), except for the first two trials, which were always presented with zero (minimal) feedback delay to allow participants to acclimate to the task. Thus, participants were exposed to 20 trials each of the non-zero delay conditions, in random order (not blocked by delay). Participants were asked to complete 2 blocks of *3SYL* sequences and 2 blocks of *4SYL* sequences in each session.

*TIMIT sentences (SENTENCES).* A set of 100 sentences and corresponding audio recordings was algorithmically selected from the Texas Instruments – Massachusetts Institute of Technology (TIMIT) continuous speech corpus [10]. The chosen sentences were subject to several constraints: 4-7 words in length; spoken duration below the 80^th^ percentile across all of TIMIT; dialects included Northern, North Midland, South Midland, New York City, Western, and “Army Brat” (indicating the speaker moved around the United States). The specific sentence sets were chosen using a custom, greedy algorithm that minimized the correlation between three parameters in the set: (i) average number of syllables per word (a proxy for word complexity), (ii) the number of consonant clusters per syllable (a proxy for articulatory complexity), (iii) and the number of syllables per second in the selected audio exemplar (speech rate).

*SENTENCES* stimuli were presented both orthographically and (simultaneously) aurally using the chosen exemplar recording (16-bit, 16 kHz sampling rate). Participants were asked to remember the short sentence and, when prompted with a visual cue, to produce it with the same rate and similar rhythm and timing as in the exemplar; subjects were specifically instructed not to try to imitate the speaker's voice. A visual “progress bar” moved from left to right, with movement duration matched to the audio exemplar duration, to help participants pace their speech appropriately. The audio recording period for each trial was set to the length of the auditory exemplar plus one second. Each experimental block consisted of 50 trials / sentences, each presented with an auditory feedback delay chosen pseudorandomly from (0, 150, 200, 250 ms). As with the *3SYL* and *4SYL* conditions, the first two trials were set to zero delay to allow participants to acclimate to the task. Participants were exposed to 16 trials each of the non-zero delay conditions, in random order (not blocked by delay). Participants were asked to complete 2 *SENTENCES* blocks in each session.

*Formant acuity (just noticeable difference) (JND).* In each session, participants completed a brief task to measure their auditory acuity to changes in the first formant frequency (*F_1_*) of their own speech using methods as described in Abur et al [15]. This measure provides an index of perceptual sensitivity to detect changes in their own speech. Participants were first asked to produce the words “bid,” “bad,” and “bed” three times each with a sustained vowel. The “bid” production with median *F_1_* values was used as the reference stimulus in the acuity (listening) task. JNDs were measured using a two-alternative forced choice (2AFC) paradigm with an adaptive staircase algorithm (2-down, 1-up) to obtain the *F_1_* difference resulting in ∼71% correct.

In this acuity task, stimuli were presented in pairs, with a 500 ms inter-stimulus gap. On 80% of trials, either the first or second stimulus (equally likely) was perturbed by the current adaptive perturbation level using Audapter software [16], while the other stimulus was the reference^2^. On the remaining 20% of trials, the two paired reference stimuli were identical (“catch trials”). Participants were asked to respond by button press if the two stimuli sounded the same or different. The initial perturbation level was set to 40% of the *F_1_* value. When the participant provided the correct answer for two consecutive trials, the perturbation level was decreased by 3%; when the participant answered any trial incorrectly, the perturbation level increased by 3%. The JND experiment ended after 10 reversals (changes in the direction of the adaptive step) or 60 trials. The participant's JND for that session was defined as the average perturbation level over the final 4 reversals.

*Spontaneous speech synchronization task (SSS).* In Session 2 (where applicable), participants completed the Spontaneous Speech Synchronization Test as described by Assaneo and colleagues [5]. This task is used to measure participants’ propensity to synchronize their speech output with an auditory input stream, revealing two groups, which were associated with neuroanatomical differences in frontal to auditory pathways [5]. Participants first listened to a stream of random syllables while whispering “ta” and were asked to adjust a knob to increase their headphone volume to a level that masked their own voice. Next, they listened to a stream of “ta” syllables produced at a rate of 4.5 syllables per second, which they were then asked to whisper at that same rate. Finally, during the main block of the SSS task, participants were asked to listen and attend to a rhythmic syllable stream presented over headphones while repeatedly whispering “ta.” They were not explicitly instructed to synchronize their whispers to the auditory stream. Subjects completed two blocks of the task, after which they were asked to report whether each of 8 syllables were present in the heard stream.

*Overall session structure.* 24 unique orders of the main tasks were created, and each participant was assigned an order for each session pseudorandomly to control for any sequencing or carryover effects between different tasks. Participants were provided short breaks between runs and water to help prevent vocal dryness and fatigue. A complete experimental session lasted approximately 90 minutes. Some participants completed additional tasks within their experimental sessions, which are not included in the current data release, and which will be described elsewhere.

**Data Processing.** Continuous audio recordings from each experimental block were semi-automatically segmented into trial level .wav files using Audacity software (www.audacityteam.org). Stereo channels (encoding microphone and headphone signals) were split and saved using a systematic file naming convention into separate folders (see *Data Description* above). Manual and automated quality assurance was performed to verify proper audio segmentation and to mark trials in which, for example, participants did not produce speech.

Reading passages were used to measure habitual speaking rate (HSR) and other individual speech features including speech rate, articulation rate (excluding pauses), median *F_0_, F_0_* interquartile range, intensity interquartile range, and working vowel space area. These measures were facilitated by using the Montreal Forced Aligner [17], seeded with the reading passage transcript and a pretrained United States English acoustic model, to align recorded audio at the word and phone levels, resulting in aligned, Praat-compatible TextGrid files. These TextGrids were then manually adjusted by the research team to correct any misalignments and/or to account for any differences (e.g., repetitions or omissions) produced by the participant. Rate, F_0_, and intensity metrics were extracted using custom Praat scripts.

To calculate working vowel space area (VSA) across each reading passage, we used a custom, semi-automated approach similar to [18] and the tutorial here [19]. Formants (*F_1_* and *F_2_*) were extracted from voiced frames within time intervals marked as vowels in the TextGrid using Praat's recommended settings for male and female voices.^3^ Intervals where estimated *F_1_* values exceeded 1200 Hz were excluded. A Gaussian Mixture Model (GMM) was estimated based on the extracted *F_1_, F_2_* pairs and used to remove outliers, defined as points in which the estimated GMM density was below the first quartile by more than 1.5 times the interquartile range. A k-means cluster analysis binned the remaining data into 12 clusters, corresponding to the 12 English vowels. A convex hull was then estimated using the centroids of the 12 clusters. The working vowel space area was estimated as the area of this convex hull. The Pearson correlation between VSA values extracted from repeated productions of the same reading passage by the same speaker in the same (first) session was *r = 0.929*, suggesting the reproducibility of this approach. Across sessions (using different reading passages), the correlation was *r = 0.749.*

Audio data from the SSS test were processed to extract the Phase Locking Value (PLV), an index of synchronization between the envelopes of the whispered “ta” syllables and the aurally presented random syllable stream. Audio recordings were first trimmed manually to limit analysis to signals of interest, then processing was performed as described in [5], including envelope extraction via the Hilbert transform, cochlea-like filtering, bandpass filtering between 3.5 and 5.5 Hz, and computation of the PLV. The PLV measure takes on values in the interval [0,1] with larger numbers indicating more consistent relative phase or synchrony.

## Limitations

There are some limitations to the dataset. While our goal was to have all participants complete two experimental sessions, 13 of 55 participants only completed one session. Thus, the amount of data available per speaker differs across participants and reduces the sample size for any cross-session comparisons that might be of interest. Our intention during participant recruitment was to obtain a representative community sample; however, the obtained sample strongly overrepresents individuals who are biologically female (44 of 55 participants). Though it has been suggested that there may be sex differences in the effects of DAF [20], this dataset may have limited utility for addressing such differences. Our experimental protocols used visual signals to encourage participants to produce speech at relatively high target rates, which is difficult under DAF. Many participants produced speech rates below the target rates, sometimes resulting in an inability to complete the production of the full stimulus during a trial. This presents trial-to-trial differences that should be carefully considered and a potential issue in interpretation since reduced speaking rates may alter the effects of DAF. However, it is possible to measure trial-to-trial speaking rates and included these as variables of interest in any analyses conducted.

## Ethics Statement

This research was carried out in accordance with the Declaration of Helsinki and United States Federal Policy regulations for the protection of human research subjects (45 CFR Part 46). Written informed consent was obtained from all participants at the time of enrollment in accordance with the University of Pittsburgh Institutional Review Board (IRB; STUDY19090083). Participants who completed a second session were verbally asked for continued consent prior to that session. The original consent asked for permission to share data through a password-protected database. With IRB approval, a follow-up survey was provided via email to ask if participants were “willing to share your de-identified study data, including audio recordings, to an *unrestricted* research database.” Data from participants who responded and provided this additional level of permission are included in the *Open Access Dataset*, while data from those who provided permission only for sharing in a password-protected database are included in the *Restricted Dataset*.

## Credit Author Statement

**Matthias Heyne:** Methodology, Software, Validation, Formal Analysis, Investigation, Data Curation, Writing – Original Draft, Writing – Review & Editing, **Monique C. Tardif:** Software, Formal Analysis, Investigation, Data Curation, Writing – Review & Editing, **Alexander Ocampo:** Software, Formal Analysis, Data Curation, Writing – Review & Editing, **Ashley P. Petitjean:** Investigation, Data Curation, **Emily J. Hacker:** Investigation, Data Curation, **Caroline N. Fox:** Investigation, Data Curation, **Megan A. Liu:** Investigation, Data Curation, **Madeline Fontana:** Data Curation, **Vincent Pennetti:** Methodology, Software, **Jason W. Bohland:** Conceptualization, Methodology, Software, Formal Analysis, Investigation, Resources, Writing – Original Draft, Writing – Review & Editing, Visualization, Supervision, Project Administration, Funding Acquisition.

## Data Availability

Open Science FrameworkDataset of speech produced with delayed auditory feedback (Original data). Open Science FrameworkDataset of speech produced with delayed auditory feedback (Original data).

## Footnotes

1 We note that the TIMIT corpus is not an open access resource but is available to many research groups. Thus, we do not provide these audio recordings in the dataset but instead a pointer to specific files in that corpus.

## Footnotes

2 The reference stimulus was (in all cases) also perturbed by 1% to control for any unexpected effects introduced by the Audapter perturbation algorithm.

3 https://www.fon.hum.uva.nl/praat/manual/Sound__To_Formant__burg____.html
